# Supplementary material for: MetaRibo-Seq measures translation in microbiomes
Source: Nat Commun. 2020 Jun 29;11:3268. doi: 10.1038/s41467-020-17081-z (PMC7324362; doi:10.1038/s41467-020-17081-z)
Supplement: Supplementary file 10 — Supplementary Data 7 [file 41467_2020_17081_MOESM10_ESM.zip › File2/Confidence_VeryHigh_Taxonomy/179141_out.krona.html]

Javascript must be enabled to view this page.

members
magnitude
magnitudeUnassigned
count
unassigned
taxon
rank

179141\_out

6

2

SRS012969\_contig\_number\_contig-100\_9073.9073SRS023526\_contig\_number\_contig-100\_43976.79559

2
superkingdom
4

1239
phylum
4

4
class
186801

4
order
186802

family
186806
3

genus
1730
3

species
142586
1

SRS050941\_contig\_number\_19398

2

SRS014613\_contig\_number\_20513SRS054905\_contig\_number\_2910
39485
species

1
family
186803

genus
841
1

species
2049040

SRS148424\_contig\_number\_contig-100\_51.195918
1
